# *Pseudomonas aeruginosa* Triggered Exosomal Release of ADAM10 Mediates Proteolytic Cleavage in Trans

**DOI:** 10.3390/ijms23031259

**Published:** 2022-01-23

**Authors:** Ahmad Aljohmani, Bastian Opitz, Markus Bischoff, Daniela Yildiz

**Affiliations:** 1 Institute of Experimental and Clinical Pharmacology and Toxicology, PZMS, ZHMB, Saarland University, 66421 Homburg, Germany; ahmad.aljohmani@uks.eu; 2 Department of Infectious Diseases and Respiratory Medicine, Charité–Universitätsmedizin Berlin, Corporate Member of Freie Universität Berlin and Humboldt-Universität zu Berlin, 10117 Berlin, Germany; bastian.optiz@charite.de; 3 Institute for Medical Microbiology and Hygiene, Saarland University, 66421 Homburg, Germany; markus.bischoff@uks.eu

**Keywords:** proteolysis, metalloproteinase, infection, exosomes, cell-cell-communication

## Abstract

Pneumonia is a life-threatening disease often caused by infection with *Streptococcus pneumoniae* and *Pseudomonas aeruginosa*. Many of the mediators (e.g., TNF, IL-6R) and junction molecules (e.g., E-cadherin) orchestrating inflammatory cell recruitment and loss of barrier integrity are proteolytically cleaved through a disintegrin and metalloproteinases (ADAMs). We could show by Western blot, surface expression analysis and measurement of proteolytic activity in cell-based assays, that ADAM10 in epithelial cells is upregulated and activated upon infection with *Pseudomonas aeruginosa* and Exotoxin A (ExoA), but not upon infection with *Streptococcus pneumoniae*. Targeting ADAM10 by pharmacological inhibition or gene silencing, we demonstrated that this activation was critical for cleavage of E-cadherin and modulated permeability and epithelial integrity. Stimulation with heat-inactivated bacteria revealed that the activation was based on the toxin repertoire rather than the interaction with the bacterial particle itself. Furthermore, calcium imaging experiments showed that the ExoA action was based on the induction of calcium influx. Investigating the extracellular vesicles and their proteolytic activity, we could show that *Pseudomonas aeruginosa* triggered exosomal release of ADAM10 and proteolytic cleavage in trans. This newly described mechanism could constitute an essential mechanism causing systemic inflammation in patients suffering from *Pseudomonas aeruginosa*-induced pneumonia stimulating future translational studies.

## 1. Introduction

Pneumonia is a major complication that often requires hospitalization and is highly associated with a substantial increase in morbidity and mortality [1]. *Streptococcus pneumoniae* (*S. pneumoniae*) and *Pseudomonas aeruginosa* (*P. aeruginosa*) are the most common causes of Gram-positive community acquired pneumonia and Gram-negative hospital acquired pneumonia, respectively [2,3]. These bacterial pathogens, which are frequently found as colonizers of the upper airways, and which can reach the lower respiratory tract by aspiration, can cause pneumonia if the pulmonary immune system is not immediately able of clearing the infection. In worst case, the initiated pneumonia leads to the development of an acute respiratory distress syndrome (ARDS) with a mortality of 43%.

Epithelial cells do not only form the first mechanical barrier against invading pathogens, but further function as “non-traditional immune cells” [4]. Their activation can be initiated either through interaction with the pathogens via Toll-like receptors (TLRs) and other pattern recognition receptors located on the cell surface, or through intracellular sensing of bacterial derivatives like DNA, cell wall fragment or the secreted toxins [5,6]. *P. aeruginosa*, as one example, is recognized by the lung epithelium through TLR2 and TLR4, thereby initiating a proinflammatory response and secretion of proinflammatory cytokines such as IL-1β IL-8, IL-6 and granulocyte-macrophage colony-stimulating factor (GM-CSF) [7,8,9,10]. Furthermore, the host cells polarity is attenuated predisposing to changes in the barrier integrity [11]. *P. aeruginosa* is equipped with several protein secretion systems, with T2SS (release of the proteases LasA/LasB and Exotoxin A (ExoA)) and T3SS (ExoS, ExoU, ExoT and ExoY) being the most prominent members [12]. ExoA, for example, exerts its ADP-ribosyltransferase activity on the eukaryotic elongation factor 2 (eEF-2) [13] and has been shown to facilitate the growth of *P. aeruginosa*, to promote lung infiltration and to disrupt the lung epithelial barrier and protein permeability by preventing the healing process of the tight junction [14,15,16]. Thus, both the bacterial particle itself and bacteria-derived toxins are able to cause pneumonia with disruption of epithelial integrity, induce leukocyte recruitment, cause alveolar edema, and stimulate release of reactive oxygen species and inflammatory mediators and impairment of alveolar gas exchange.

Many of the mentioned factors and disease mechanisms are common amongst inflammatory and infectious diseases and have been shown to be influenced by members of the a disintegrin and metalloproteinase (ADAM) family. ADAMs are transmembrane zinc-dependent metalloproteinases of a multi-domain structure. The release of the soluble ectodomain by ADAM proteases (shedding) has been shown for a high number of transmembrane molecules such as growth factors (e.g., heparin-binding EGF-like growh factor (HB-EGF)), cytokines and chemokines (e.g., TNF), their receptors (e.g., TNFR, IL-6R) and adhesion molecules (E- and VE-Cadherin) [17]. Several studies have reported that ADAM proteases, especially ADAM10 and ADAM17, contribute to the inflammatory response through regulation of pathogen recognition and entrance, toxin handling, phagocytosis and clearance, cytokine release, leukocyte recruitment, local and systemic changes, resolution of inflammation and regeneration (for review, see [18]). In previous studies we could show that ADAM10 in leukocytes is crucial for recruitment of leukocytes in sterile lung inflammation [19]. On the other hand, ADAM17 deficiency enhanced the phagocytic capacity of leukocytes in a cell-autonomous manner [20]. For endothelial cells, it was shown that TLR4 shedding by ADAM10 reduced the inflammatory responsiveness to lipopolysaccharide (LPS) [21]. In epithelial cells, ADAM10 acts as a receptor for *Staphylococcus aureus* (*S. aureus*) pore-forming α-hemolysin, promoting E-cadherin shedding and disruption of the barrier integrity [22,23]. Based on previous studies, one could assume that ADAM10 fulfills different functions in a pathogen-dependent manner. However, systematic comparisons are lacking so far.

Here, we aimed to provide evidence for a pathogen-dependent regulation and function of ADAM10 in lung epithelial cells using the most common pathogens causing bacterial pneumonia, *P. aeruginosa* and *S. pneumoniae*, respectively. We could show that ADAM10 is upregulated and activated upon infection with *P. aeruginosa* and one of its major virulence factors ExoA, but apparently not upon encounter with *S. pneumoniae*. This activation of ADAM10 in *P. aeruginosa* infection was critical for cleavage of the junction molecule E-cadherin and contributed to induction of permeability, with ADAM10 affecting the initial damage of the epithelial barrier rather than wound closure. The pathogen-dependent activation was based on the toxin repertoire and induction of calcium signaling processes through calcium influx in case of ExoA rather than the interaction with the particle itself. Most importantly, infection with *P. aeruginosa* resulted in the release of ADAM10 on exosomes, mediating proteolytic cleavage in trans (on distinct cells). Thus, we could provide evidence for a pathogen-dependent activation of ADAM10 during bacterial infection. This *P. aeruginosa*-triggered exosomal release of ADAM10 could constitute an essential mechanism of the pathogen to cause systemic inflammation in patients suffering from *P. aeruginosa*-induced pneumonia stimulating future preclinical and translational studies.

## 2. Results

### 2.1. ADAM10 Is Pathogen-Specifically Regulated through Infection of Lung Epithelial Cells

**Figure 1:**
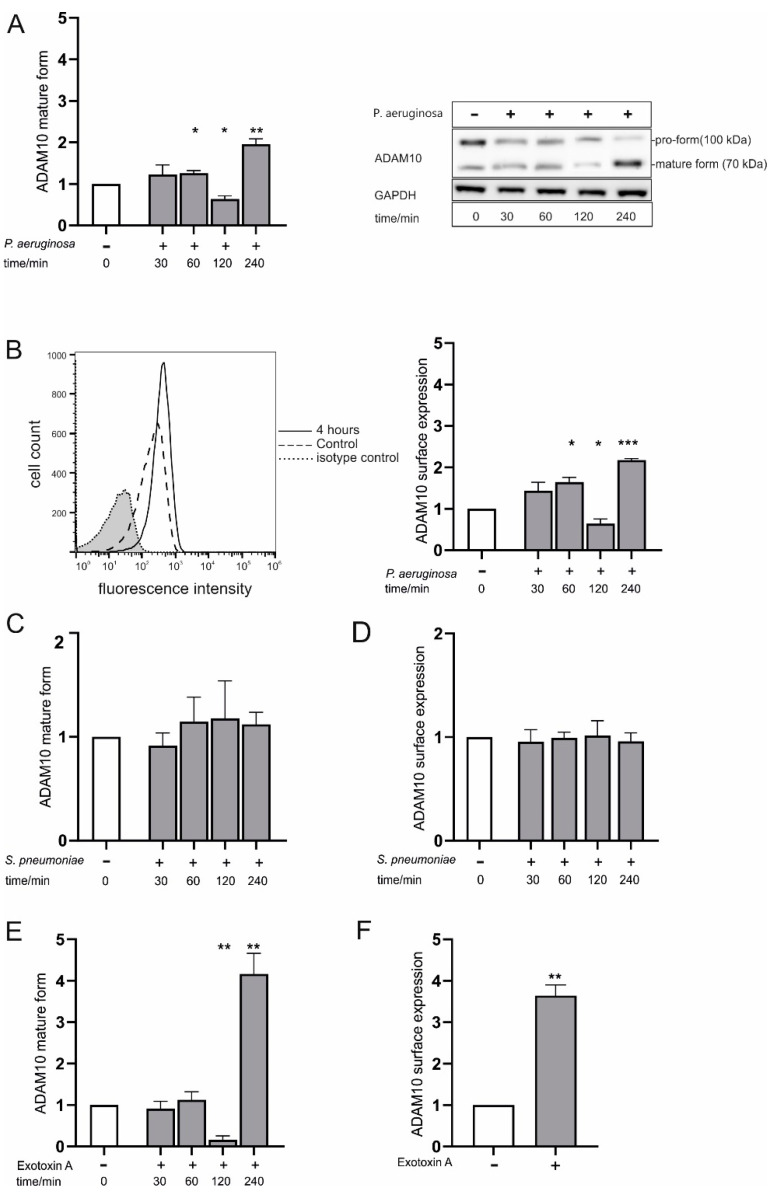
Pathogen-specific regulation of a disintegrin and metalloproteinase (ADAM)10 protein expression and surface localization in bacterial infection. A549 cells were grown to confluence and either left unstimulated or infected with *Pseudomonas aeruginosa (P. aeruginosa*) (multiplicity of infection of 5 (MOI 5) (**A**,**B**), infected with *Streptococcus pneumoniae* (*S. pneumoniae*) (MOI 5) (**C**,**D**) or stimulated with exotoxin A (ExoA) (100 ng/mL, **E**,**F**). In (**A**–**E**), samples were taken after an incubation time of 30, 60, 120 or 240 min. In (**F**), samples were probed after 4 h. (**A**,**C**,**E**): ADAM10 protein expression and maturation was investigated in cell lysates by Western blot probing with an antibody against the C-terminus (intracellular part). Probing against glyceraldehyde-3-phosphat dehydrogenase (GAPDH) served as loading control. Band intensities of the pro-form (100 kDa) and the mature form (70 kDa) were quantified by densitometry and normalized to the expression of the unstimulated cells (0 h). A representative blot is shown in (**A**, right) (antibody specificity detailed in Figure S1). (**B**,**D**,**E**): ADAM10 surface expression was investigated by surface staining with an N-terminal antibody against ADAM10 (1 µg/mL) and an APC-coupled secondary antibody (5 µg/mL) and subsequent flow cytometric analysis (quantification as mean fluorescence intensity). The values of the adequate isotype control were subtracted followed by normalization to the unstimulated cells. A representative histogram is shown in (**B**, left). Quantitative data are shown as means + SD of three independent experiments. Asterisks indicate significance difference to the control calculated using two tailed two samples t-test (* *p* < 0.05, ** *p* < 0.01, *** *p* < 0.001).

Pneumonia is characterized by damage of the alveolar epithelium, which belongs to the first line defense against pathogens. Here, we investigated the regulation of ADAM10 in A549 cells (alveolar adenocarcinoma cells) and human small airway epithelial cells (HSAEpC) during infection and sterile inflammation using Western blot (cell lysates) and analysis of surface expression on intact cells. In A549 cells, the Gram-negative bacterium *P. aeruginosa* promoted maturation of ADAM10, indicated by an increase of the mature form (70 kDa) and a decrease in the pro-form (100 kDa), after 1 and 4 h of infection (Figure 1A, supplemental figure (Figure S1A)).

Interestingly, the mature form of ADAM10 was vanished in the cell lysate after 2 h of infection, while the pro-from stayed constant. The same increase and drop were observed for surface expression of ADAM10, investigated by flow cytometry (Figure 1B). As shown below, we explain this 2 h drop by intermediate release of ADAM10 from the surface on extracellular vesicles. In contrast, infection with the Gram-positive bacterium *S. pneumoniae* exerted no impact on ADAM10 protein expression/maturation (Figure 1C and Figure S1B) and surface localization (Figure 1D). Stimulation might not only occur through contact with the pathogen itself, but also via interaction with bacterial virulence factors. The time-response curve of ADAM10 expression/maturation upon stimulation with 100 ng/mL ExoA, one of the most virulent toxins secreted by *P. aeruginosa* [14], showed the same regulation of ADAM10 expression/maturation pattern as observed by *P. aeruginosa* infection (Figure 1E, compare to Figure 1A). Further, again an up-regulation of ADAM10 surface expression was observed (Figure 1F). In comparison to A549 cells, HSAEpC challenged with *P. aeruginosa* and ExoA, respectively, showed a weaker expression of ADAM10 and a prolonged response (Figure S1C,D). Again, *S. pneumoniae* had no impact on the regulation of ADAM10 (Figure S1E). Both findings suggest that epithelial ADAM10 is regulated in a pathogen-specific manner during infection.

### 2.2. P. aeruginosa and ExoA Promote ADAM10 Activation and Shedding Activity

**Figure 2:**
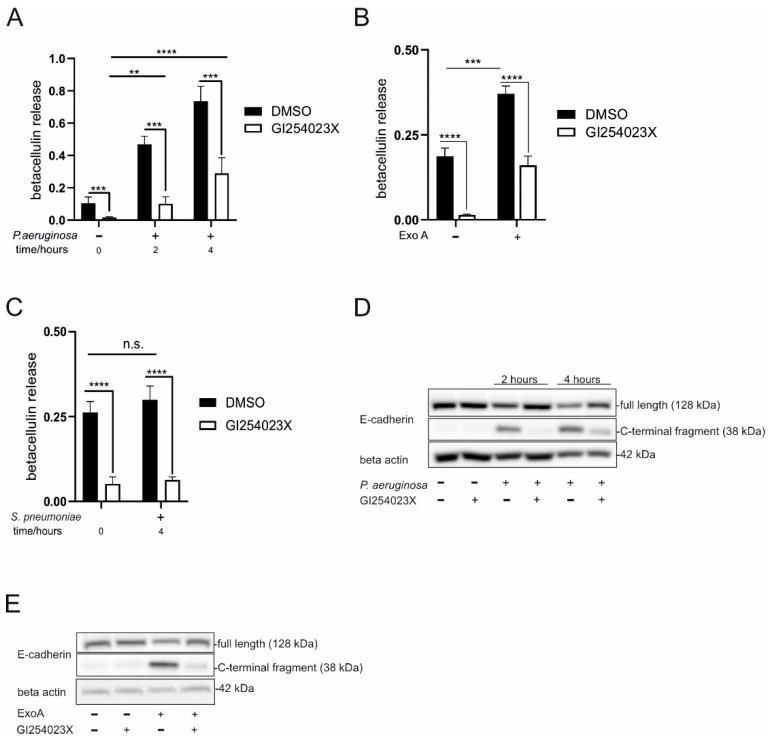
*P. aeruginosa* and ExoA promote ADAM10 activation and shedding activity. (**A**–**C**) A549 cells were transfected with a plasmid encoding for alkaline phosphatase (AP)-coupled betacellulin (AP-BTC) and seeded at equal density. Cells were pre-incubated with ADAM10 inhibitor GI254023X (10 μM) or 0.1% DMSO (vehicle control) for 30 min. Subsequently, cells were left unstimulated or infected with *P. aeruginosa* in (**A**) (MOI 5 for 2 and 4 h), stimulated with ExoA in (**B**) (100 ng/mL for 4 h), or infected with *S. pneumoniae* in (**C**) (MOI 5 for 4 h). Finally, AP activity was determined in the cell lysate and supernatant to quantify the relative betacellulin cleavage and release. (**D**,**E**) A549 cells were either pre-incubated with ADAM10 inhibitor GI254023X (10 μM) or 0.1% DMSO (vehicle control) for 30 min. Subsequently, cells were left unstimulated and either infected with *P. aeruginosa* in (**D**) (MOI 5 for 2 and 4 h) or stimulated with ExoA in (**E**) (100 ng/mL, for 4 h). After the mentioned stimulation time, cells were lysed and cleavage of E-cadherin was investigated by Western blot, probing with antibodies against the C-terminus of E-cadherin followed by beta actin as loading control. Representative blots of three independent experiments are shown. Quantitative data (**A**–**C**) are shown as means + SD of three independent experiments. Asterisks indicate significance among treated cells calculated using two-way ANOVA and Tukey post-test (** *p* < 0.01, *** *p* < 0.001, **** *p* < 0.0001, n.s. not significant).

To investigate the functional impact of surface expressed ADAM10 in these settings, we next studied the activity of ADAM10 in response to *P. aeruginosa* and ExoA by cleavage of alkaline phosphatase (AP)-tagged betacellulin (AP-BTC) [24] and the endogenous substrate E-cadherin. Both betacellulin and E-cadherin have been described as ADAM10 specific substrates [25,26]. Time points were chosen according to the observed changes in maturation. As the prolonged response of HSAEpC would result in bacterial overgrowth thereby limiting the use in mechanistic and functional studies, we went on with investigations in A549 cells. Infection with *P. aeruginosa* resulted in a significant increase of AP activity in the cell supernatant after 2 and 4 h in comparison to control (Figure 2A), showing a correlation with enhanced betacellulin shedding. Notably, this release was comparable to stimulation with ionomycin as one of the strongest activators of ADAM10 (Figure S2A) and significantly decreased by pre-treatment with the ADAM10 specific inhibitor GI254023X (GI) (Figure 2A). Furthermore, GI reduced the basal betacellulin release observed in control cells. Moreover, ExoA significantly induced betacellulin release, which could be significantly attenuated by GI pretreatment (Figure 2B). Despite the lack of changes in ADAM10 expression patterns upon infection with *S. pneumoniae*, differences might occur on the level of activation. However, infection with *S. pneumoniae* did not change the activity of ADAM10, remaining on the basal level of non-stimulated control cells (Figure 2C). Next, we investigated the shedding of E-cadherin as endogenous substrate. Both infection with *P. aeruginosa* and stimulation with ExoA induced shedding of E-cadherin indicated by an increase in the C-terminal fragment (38 kDa) and a decrease in the full-length protein (128 kDa), detected by the same antibody, while pharmacological inhibition of ADAM10 clearly decreased the cleavage (Figure 2E,F). Thus, ADAM10 maturation and translocation to the surface resulted in increased shedding activity.

### 2.3. Epithelial ADAM10 Contributes to P. aeruginosa and ExoA Induced Changes in Permeability

**Figure 3:**
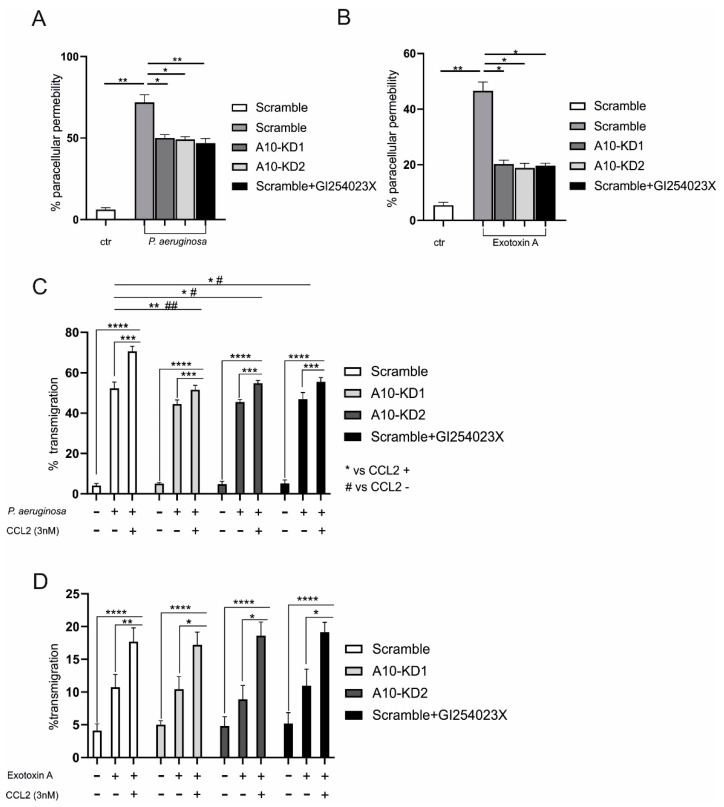
Function of ADAM10 in *P. aeruginosa* induced protein permeability and leukocyte transmigration. A549 cells were transduced with lentivirus encoding shRNA against ADAM10 for knockdown (KD) (A10-KD1 or 10-KD2) or an unspecific shRNA (scramble, scr) as indicated in the graph legend. Cells were grown in trans wells until confluence. Cells were pre-incubated with 0.1% DMSO (vehicle control) or with ADAM10 inhibitor GI254023X (10 μM, black bars). (**A**,**B**) Cells were either left unstimulated or infected with *P. aeruginosa* in (**A**) (MOI 5 for 4 h) or stimulated with ExoA in (**B**) (100 ng/mL for 4 h). Subsequently, the cell culture medium in the upper chamber was replaced by 70-kDa TRITC dextran and FITC-albumin suspension in PBS supplemented with 0.2 % BSA, and the permeability was measured by TRITC-dextran and FITC-albumin diffusion into the lower wells. Paracellular permeability is shown as percentage, calculated in relation to the background empty transwell (100%). (**C**,**D**) Cells were either left unstimulated, infected with *P. aeruginosa* in (**C**) (MOI 5 for 4 h) or stimulated with ExoA in (**D**) (100 ng/mL for 4 h). Subsequently, 2 × 10^5^ THP-1 cells were added to the upper chamber in the presence or absence of human CCL-2 (3 nM) as chemoattractant for monocytes. After 45 min, the number of transmigrated cells was determined by measurement of endogenous β-glucoronidase activity in the lower chamber. Quantitative data are shown as mean + SD of three independent experiments. Asterisks indicate significance among treated cells calculated using one-way ANOVA and Tukey post-test in (**A**,**B**). In (**C**,**D**), asterisks and rhombs indicate significance among treated cells in the absence or presence of CCL2, respectively, calculated using two-way ANOVA and Tukey post-test (*/# *p* < 0.05, **/## *p* < 0.01, *** *p* < 0.001, **** *p* < 0.0001).

E-cadherin and other junction and adhesion molecules contribute to barrier integrity [26,27], thereby promoting changes of tissue permeability as clinical manifestations of pneumonia. Therefore, A549 monolayers grown on transwells were infected with *P. aeruginosa* and stimulated with ExoA for 4 h (time point of maximal ADAM10 response), respectively, and investigated for paracellular and total (paracellular plus transcellular) protein permeability using TRITC-dextran and FITC-albumin as tracers, respectively. Both *P. aeruginosa* infection and stimulation with ExoA significantly increased the paracellular and total (paracellular and transcellular) permeability in comparison to control-treated cells (Figure 3A,B and Figure S3A,B). This effect was suppressed by either treatment with GI or gene silencing/knockdown upon transduction with lentiviral particles carrying ADAM10-specific shRNAs (knockdown efficiency controlled by Western blot, see Figure S1A).

Lung epithelial cells and macrophages belong to the first line defense against invading pathogens. We next examined whether ADAM10 activity may influence transepithelial migration of THP-1 cells during infection with *P. aeruginosa*. Infection with *P. aeruginosa* (4 h) significantly increased the transepithelial migration of THP-1 in comparison to control treated cells (Figure 3C, white bars). This effect was observed both in the presence (14-fold) and absence (10-fold) of CCL2 as a monocyte chemoattractant. Nevertheless, CCL-2 led to further increase of transmigration, which was abrogated by both gene silencing of ADAM10 or a concomitant incubation with GI (reduction to 10-fold). In addition, both ADAM10 targeting approaches reduced the transmigration solely caused by *P. aeruginosa* (from 10-fold to eight-fold), whereas no effect on random transmigration was observed (Figure 3C). Stimulation with ExoA showed a much weaker induction of transmigration in comparison to infection with *P. aeruginosa* (only appr. 20%). However, no effect of ADAM10 inhibition or gene silencing was observed (Figure 3D). Thus, *P. aeruginosa* may induce changes in cell permeability through activation of ADAM10, which in parts effects leukocyte transmigration.

### 2.4. ADAM10 Inhibition Improves Epithelial Wound Healing

**Figure 4:**
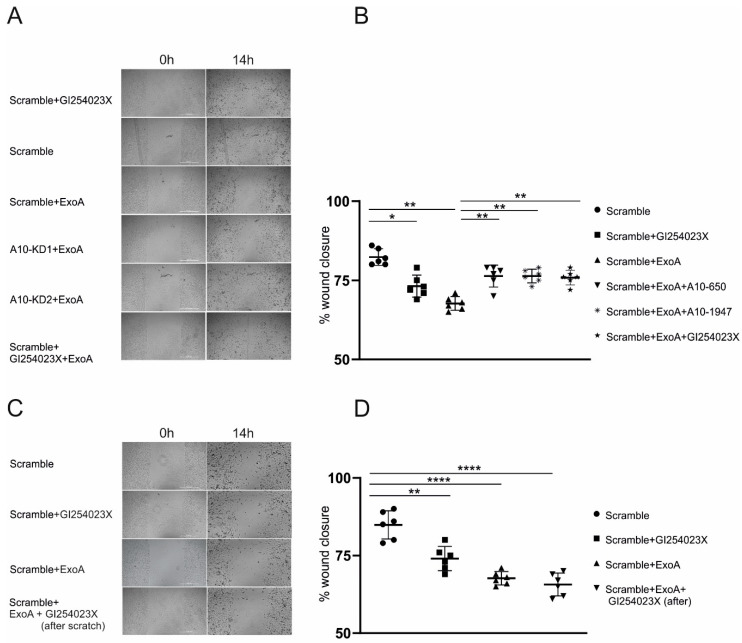
Differential function of ADAM10 in epithelial regeneration. A549 cells were transduced with lentivirus encoding shRNA against ADAM10 for knockdown (KD) (A10-KD1 or 10-KD2) or an unspecific shRNA (scramble, scr), as indicated in the graph legend. Cells were grown in 96-well plates until confluence and treated for 2 h with mitomycin (5 µg/mL) to avoid cell proliferation. In (**A**,**B**), cells were pre-incubated with 0.1% DMSO (vehicle control) or with ADAM10 inhibitor GI254023X (10 μM). Subsequently, cells were either left unstimulated or stimulated with ExoA (100 ng/mL). After 4 h, the stimulant was removed and the cells were investigated for wound closure after automated scratch induction over a period of 24 h using a live cell imaging system. In (**C**,**D**), the same setup was used. Additionally, GI254023X was added after ExoA treatment and scratch induction. Data are shown as a percentage of wound closure relative to control treated cells (**A**,**C** representative images; **B**,**D** automated quantifications). Quantitative data are shown as means + SD of six independent experiments. Asterisks indicate significance among treated cells calculated using one-way ANOVA and Tukey post-test (* *p* < 0.05, ** *p* < 0.01, **** *p* < 0.0001).

Severe pneumonia is not only associated with increased permeability and transepithelial migration of leukocytes, but may further result in strong epithelial damage. Therefore, we investigated the effect of ADAM10 activation upon stimulation with ExoA in a scratch wound closure assay in the presence and absence of GI. Inhibition of ADAM10 led to a reduction of wound closure by appr. 10%. Stimulation with ExoA impaired wound healing of A549 cells compared to the control by appr. 20%. Interestingly, concomitant inhibition of ADAM10 did not result in a synergistic effect but improved the wound closure comparing to ExoA stimulated cells to levels of single GI treatment (Figure 4A,B). The same results were observed when the experiments were performed upon gene silencing of ADAM10 instead of inhibition. These findings suggest that ExoA induces an ADAM10-dependent cell damage leading to impaired migration. To test this hypothesis in more detail, the experiments were repeated without GI preincubation and addition only in the migration/wound closure phase (after stimulation with ExoA). Inhibition of ADAM10 during the migration phase had no additional effect on epithelial cell migration, indicating a role of ADAM10 in mediating cell damage rather than the cellular migration (Figure 4C,D). Thus, ADAM10 may fulfill several pro-inflammatory functions during infection by orchestrating protein permeability, transepithelial migration as well as epithelial damage and regeneration/migration.

### 2.5. P. aeruginosa Induces Exosomal Release of ADAM10

**Figure 5:**
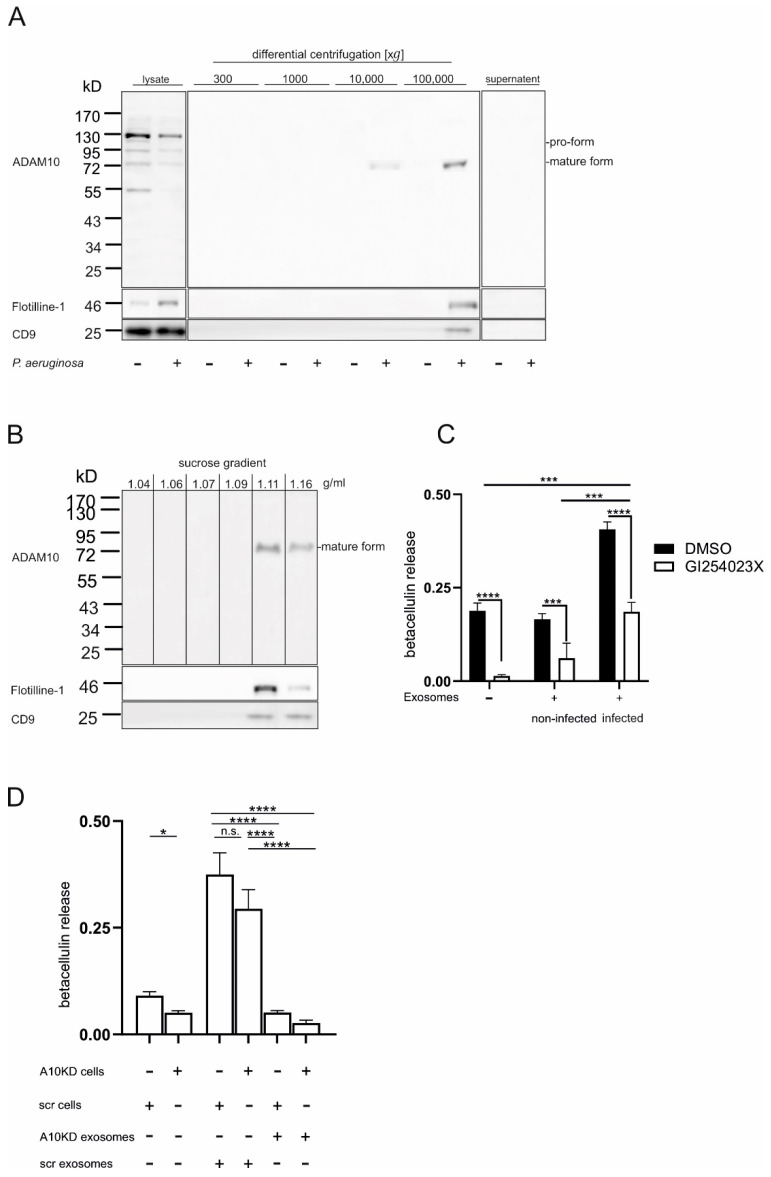
Functional implication of exosomal ADAM10 *P. aeruginosa* infection. (**A**) The 2 × 10^7^ A549 cells were left unstimulated or infected with *P. aeruginosa* (MOI 5) in serum free medium for 2 h. Subsequently, the cell supernatant was subjected to differential centrifugation (300, 1000, 10,000, 100,000 *g*). The pellets obtained in each sequential centrifugation step and the cells were lysed and subjected to Western blot analysis together with the unfractionated supernatants. Membranes were probed against ADAM10 (C-terminal antibody), Flotiline-1 and CD9 (positive markers for exosomes). (**B**) Extracellular vesicles from *P. aeruginosa* infected cells were collected as described in A and subjected to sucrose density gradient centrifugation after centrifugation at 100,000 *g*, Purity of fractions was controlled by optical density measurements, and fractions were subjected to Western blot analysis. Membranes were probed against ADAM10, Flotiline-1 and CD9. Notably, the ADAM10 positive fractions were identified as exosomes by the exosomal markers and the density. In (**A**,**B**), representative blots of at least three independent experiments are shown. (**C**) A549 cells were transfected with a plasmid encoding for AP-BTC and seeded at equal density. Cells were pre-incubated with ADAM10 inhibitor GI254023X (10 μM) or 0.1% DMSO (vehicle control) for 30 min. Subsequently, cells were left untreated (no exosomes) or co-incubated with exosomes derived from either non-infected or *P. aeruginosa* infected (MOI 5 for 2) cells. After 2 h, AP activity was determined in the cell lysate and supernatant to quantify the relative betacellulin cleavage and release. (**D**) A549 cells were transduced with lentivirus encoding shRNA against ADAM10 for knockdown (KD) (A10-KD1 or 10-KD2) or an unspecific shRNA (scramble, scr) as indicated in the graph legend. Cells for activity measurement were transfected with a plasmid encoding for AP-BTC prior to seeding. Cells for exosome preparation were not transfected. Cells were either left untreated or co-incubated with exosomes prepared from *P. aeruginosa* infected (MOI 5 for 2 h) scramble or A10 KD cells, respectively. After 2 h, AP activity was determined in the cell lysate and supernatant to quantify the relative betacellulin cleavage and release. Quantitative data are shown as means + SD of three independent experiments. Asterisks indicate significance among treated cells calculated using two-way ANOVA and Tukey post-test in (**C**) and one-way ANOVA and Tukey post-test in (**D**) (* *p* < 0.05, *** *p* < 0.001, **** *p* < 0.0001, n.s. not significant).

Interestingly, infection of A549 cells with *P. aeruginosa* for 2 h resulted in ADAM10 activation although the presence of mature ADAM10 was reduced on the cell surface as well as in the cell lysates. ADAM10 has been shown to be present and active in exosome like extracellular vesicles (EVs) (e.g., derived from Hodgkin lymphoma and ovarian carcinoma cells) [28,29]. Therefore, we hypothesized that *P. aeruginosa* could induce an exocytotic release of ADAM10 to the extracellular environment and investigated the EV fraction originating from *P. aeruginosa* infected and non-infected A549 cells for ADAM10 expression. Mature ADAM10 was enriched in the vesicle fraction obtained by centrifugation at 100,000 *g* after stimulation with *P. aeruginosa* while it was totally absent in the non-infected cells (Figure 5A). This vesicle fraction was further characterized as flotillin-1 and CD9 positive. Only week expression was observed in the 10,000 g fraction. It is important to note that no CD9 and flotiline-1 containing vesicles could be observed in non-infected cells in the investigated time frame, indicating a massive increase in EV release upon infection. To further classify the EVs derived from *P. aeruginosa* infected cells, EVs were subjected to sucrose density gradient centrifugation to fractionate the vesicles according to their density. Expression of ADAM10, flotillin-1 and CD9 was seen at a density of 1.11 and 1.16 g/mL, respectively, which represent the typical density of exosomes (Figure 5B). These observations suggest that infection with *P. aeruginosa* induces the release of mature ADAM10 in exosomes.

### 2.6. P. aeruginosa Triggers ADAM10-Mediated Cleavage in Trans

We next questioned if the observed increase in ADAM10 activity may result from ADAM10 in exosomes. Therefore, A549 cells transfected with AP-BTC were treated with exosomes derived from unstimulated or *P. aeruginosa* infected cells (not bearing AP-BTC). Interestingly, only exosomes derived from *P. aeruginosa* infected cells were able to induce the cleavage of betacellulin and the release of AP into the supernatant. This effect could be blocked by pharmacological inhibition of ADAM10 using GI (Figure 5C). Thus, exosomal ADAM10 is active and mediates the ectodomain shedding of BTC on the cell surface of distinct cells, indicating a cleavage in trans. To exclude that remaining toxin or other bacterial products in the exosome preparation would stimulate ADAM10 on the cell surface and to ensure that ADAM10 activity was of exosomal nature, we purified exosomes from ADAM10 knockdown (KD) cells and the respective control cells after infection with *P. aeruginosa*. These exosomes were added to AP-BTC transfected cells to evaluate the cleavage of BTC. Exosomes derived from ADAM10 expressing cells induced a significant cleavage of BTC on both control and ADAM10 KD cells (Figure 5D). In contrast, exosomes derived from ADAM10 KD cells had no effect on both cell types. Thus, *P. aeruginosa* induces exosomal release of active ADAM10 mediating ectodomain shedding on distinct cells (in trans).

### 2.7. The Pathogen-Specific Activation of ADAM10 in P. aeruginosa Infection Depends on the Secreted Toxins and Intracellular Calcium Increase in Case of ExoA

**Figure 6:**
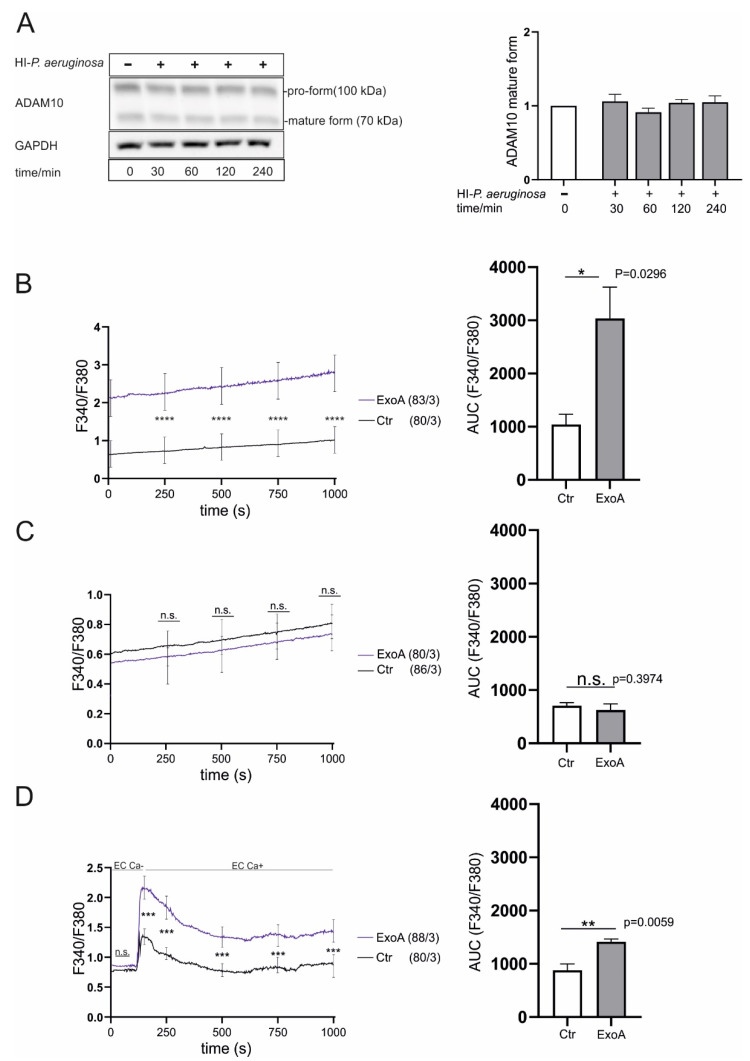
Pathogen-specific activation of ADAM10 depends on the toxin repertoire and calcium increase. (**A**) A549 cells were grown to confluence and either left unstimulated or infected with heat-inactivated *P. aeruginosa* (multiplicity of infection of 5 (MOI 5). Samples were taken after an incubation time of 30, 60, 120 or 240 min. ADAM10 protein expression and maturation was investigated in cell lysates by Western blot probing with an antibody against the C-terminus (intracellular part). Probing against GAPDH served as loading control. Band intensities of the pro-form (100 kDa) and the mature form (70 kDa) were quantified by densitometry and normalized to the expression of the unstimulated cells (0 h). A representative blot is shown in (**A**). (**B**,**C**,**D**) A549 cells were grown to 70% confluency on poly-L-lysine coated glass coverslips and either left unstimulated (PBS) or stimulated with ExoA (100 ng/mL) for 4 h in Tyrode’s solution (**B**), calcium free Tyrode’s solution (**C**,**D**). Subsequently, the cells loaded with 5 μM Fura-2 AM for 45 min at 37 °C followed by calcium signaling recording over 1000 s in Tyrode’s solution (**B**), calcium free Tyrode’s solution (**C**) or calcium free Tyrode’s solution followed by addition of 2 mM calcium after 120 s of calcium signaling recording (**D**). Quantitative data are shown as means + SD of three independent experiments. Asterisks indicate significance difference among treated cells at the indicated time point calculated using two-way ANOVA and Bonferroni post-test for F340/380 ratio over time and one-way ANOVA and Tukey post-test for area under the curve (* *p* < 0.05, ** *p* < 0.001, *** *p* < 0.001, **** *p* < 0.0001, n.s. not significant). In (**A**), significance was analyzed by two tailed two samples t-test. No significant differences were observed.

As mentioned above, cell-pathogen and cell-toxin interaction might be responsible for the observed effects on ADAM10 expression and activation. To analyze the contribution of the particle itself, *P. aeruginosa* cultures were subjected to heat-inactivation. We used mild conditions (40 min, 70 °C) to ensure toxin degeneration and bacterial death (controlled by lack of colony formation after 24 h, Figure S3C) without total disruption of the bacterial particle structure (controlled by microscopy) [30]. Notably, exposition of A549 cells to heat-inactivated *P. aeruginosa* did not change expression or maturation of ADAM10 (Figure 6A), contrary to our observations made with viable cells (Figure 1). ExoA mainly acts through inhibition of e-EF2 [31]. However, based on the known activation of ADAM10 through intracellular calcium increase, we next asked if the observed activation of ADAM10 through ExoA might be dependent on induction of calcium signaling. Indeed, calcium imaging experiments revealed an increase of intracellular calcium levels [Ca^2+^]_i_ upon stimulation with ExoA (Figure 6B). An increase of [Ca^2+^]_i_ can be caused by release from intracellular storages (e.g., endoplasmatic reticulum) or influx from outside to inside the cell. To address this point, the experiments were repeated in the absence of extracellular calcium and with addition of extracellular calcium after stimulation with ExoA, respectively. In the absence of extracellular calcium, no increase in [Ca^2+^]_i_ upon stimulation with ExoA was observed (Figure 6C). However, upon addition of extracellular calcium, a peaked increase in [Ca^2+^]_i_ was detected (Figure 6D). Thus, the pathogen-specific activation of ADAM10 through infection with *P. aeruginosa* is rather dependent on the pathogen’s toxin repertoire than the particle itself and might be triggered by intracellular calcium increase through calcium influx.

## 3. Discussion

Our present study shows that epithelial ADAM10 is involved in the inflammatory manifestations in response to *P. aeruginosa* infection and ExoA stimulation by modulating transepithelial leukocyte migration, protein permeability and epithelial regeneration. In contrast, no influence by *S. pneumoniae* was observed. This pathogen-specific regulation of ADAM10 might be predominantly depending on the secreted toxin repertoire of the pathogen and the induction of calcium influx in the case of ExoA rather than interaction with the bacterial particle itself. Furthermore, *P. aeruginosa* induced an exocytic release of active ADAM10 in exosomes to the extracellular environment resulting in proteolytic cleavage in trans which means on a distinct cellular surface.

Several pathogens and/or their secreted toxins can cause pneumonia which can lead to ARDS and sepsis. Several studies highlighted the importance of ADAM10’s proteolytic activity in sterile inflammation [19,32]; however, the regulation especially in epithelial cells in the context of lung infection is poorly addressed. It has been shown that ADAM10 itself is able to cleave TLR4 and the receptor for advanced glycation end products [21,33], which could lead to the assumption of a Gram-dependent and thus cell wall compound-dependent activation of ADAM10. For *S. aureus* it was shown that ADAM10 acts as receptor for α-hemolysin disrupting the barrier integrity and could, therefore, have a general role in bacterial pathogenesis [23]. Interestingly, *S. pneumoniae* as another Gram-positive bacterium did not have any effect on ADAM10 protein expression or proteolytic activity, whereas the Gram-negative bacterium *P. aeruginosa* stimulated ADAM10 activity and related pathogenic processes, including disruption of the epithelial barrier. Furthermore, stimulation with heat-inactivated *P. aeruginosa* particles did not induce any change of ADAM10 expression or activation. Therefore, we suppose that the pathogen-specific activation of epithelial ADAM10 depends primarily on the secreted toxin repertoire and occurs mostly independent of the interaction with pattern recognition receptors.

For *P. aeruginosa* it has been shown that infection is followed by subsequent increase of calcium influx [34], which could be due to pore-forming toxins such as exolysin A. However, calcium-imaging experiments revealed an increase of intracellular calcium upon stimulation with ExoA, which functions as ADP-ribosyltransferase inactivating eEF-2 [31]. Although the crossing of membranes is facilitated by its six α-helices, it is rather unlikely that ExoA exerts a similar pore-formation as observed for *S. aureus* α-hemolysin [22,35]. Nevertheless, we could show that the increase of intracellular calcium levels was triggered by calcium influx rather than release from intracellular storages. This is evidenced by previous studies showing an inhibitory action on calcium efflux from the endoplasmic reticulum through interaction with Sec61 [36]. ExoA enters the cell through interaction with its receptor CD91 and the subsequent uptake via either clathrin-coated pits or the lipid-sorting pathway (for review see [14]). Thus, changes of the membrane fluidity and reorganization in membrane microdomains (e.g., lipid rafts) or the activation of G-protein coupled receptors could result in the activation of calcium ion channels [37,38,39]. However, this is speculative at this stage, stimulating future studies.

After removal of the pro-domain, mature ADAM10 translocates to the surface as dimers in which the catalytic domain is blocked due to an autoinhibitory conformation. Thus, detection of mature ADAM10 is not directly related to catalytic activity of the protease (for review see [40]). However, by pharmacological inhibition and gene silencing we could clearly show that not only *P. aeruginosa* but also ExoA alone led to ADAM10-dependent cleavage of E-cadherin as endogenous substrate. Tight and adherence junction molecules work together to block the leakage of integral membrane proteins and preserve the cell polarity [41]. On the one hand, E-cadherin cleavage results in loss of junctions in epithelial cells, resulting in enhanced wound closure [26]. Indeed, inhibition of ADAM10 impaired the epithelial regeneration. ExoA has been shown to alter the epithelial integrity and to impair repair processes [42], which is in line with our findings presented here. The general impairment of wound repair in response to *P. aeruginosa* is in part regulated through ERK/p38 (MAPK) signaling pathways and the induction of reactive oxygen species [43], and an imbalance of pro-forms and activated forms of matrix metalloproteinases (MMPs) such as MMP2 and MMP9 [44,45]. For α-hemolysin, it was shown that ADAM10 is critical for the activation of the NLRP3 inflammasome [46]. In traumatic brain injury, inhibition of Adam10 attenuated the upregulation of *Mmp2* and *Mmp9* expression [47]. Furthermore, the ADAM10 and TIMP-1 complex is responsible for cell binding and processing of pro-MMP9 [48]. Thus, the improvement of wound repair by co-inhibition of ADAM10 during ExoA stimulation might be explained by the inhibition of such downstream effects, whereas the reduced wound closure by pharmacological inhibition in general and after the stimulation with ExoA depends on the reduced cleavage of E-cadherin.

Disruption of barrier integrity does not only explain the observed increase in protein permeability by us and others [15], but is also one prerequisite for systemic invasion of the pathogen itself as well as reverse migration of alveolar macrophages and leukocyte immigration into the inflamed lung. In the present study, we observed a different extent of ADAM10 contribution to transepithelial migration in response to infection with *P. aeruginosa* and stimulation with ExoA, respectively. *P. aeruginosa* with intact pilli leads to upregulation of integrin and adhesion molecules (e.g., ICAM-1) [49,50], which are essential for the adhesion of leukocytes to the inflamed tissue and the subsequent transmigration, which would be one explanation for the strong induction of transepithelial migration upon infection with *P. aeruginosa*. Further, the used strain (PA103) is known to produce high amounts of ExoA and to exert a cytotoxic phenotype also dependent on ExoU [51]. Furthermore, besides ExoA and ExoU, other released virulence factors or secretion systems such as LasB, pyocyanin and T3SS may contribute to ADAM10-dependent changes in epithelial integrity, which is also reflected on the level of paracellular permeability. This synergistic effect of different exotoxins is strengthened by the higher induction of ADAM10 activity upon *P. aeruginosa* infection compared to ExoA stimulation. However, to decipher the single contribution of each exotoxin to ADAM10 activation is far beyond the scope of the current study.

ADAM10 is released on exosomes and might be further proteolytically cleaved by other proteases and released as soluble ectodomain [28,29,52]. However, only a few studies have proposed a cleavage in trans (proteolytic cleavage on a distinct cell surface) so far [53]. In the present study, a significant amount of the measured ADAM10 activity was attributed to ADAM10 released on exosomes. We could clearly show that ADAM10 on exosomes is proteolytically active and cleaving substrates on other cells (in trans). At the current stage, we cannot exclude that the exosomes in our transfer experiments are first fusing to the plasma membrane, and subsequently cleavage would again occur in cis. However, the rate of uptake is very limited and in the observed time frame (max. 2 h) a maximal uptake of 20% would be expected [54] indicating a cleavage in trans via exosomal ADAM10 triggered by *P. aeruginosa* infection based on the observed cleavage levels. Exosomes represent a platform for cellular communication and signaling in the human body. Based on the stability of exosomes in the blood system [55], *P. aeruginosa* triggered release of exosomes upon local infection in the lung could result in systemic effects either through spreading of the shedding products (inflammatory and pro-inflammatory mediators) or transporting the proteases to other tissues to mediate the proteolytic activity [56].

This *P. aeruginosa*-triggered exosomal release of ADAM10 could constitute an essential mechanism of the pathogen to cause systemic inflammation in patients suffering from *P. aeruginosa*-induced pneumonia. To prove this hypothesis, translational studies isolating EVs from patient serum samples with microbiologically characterized infection will have to be performed, additionally elucidating the use of exosomal ADAM10 as a predictive marker in *P. aeruginosa* infection. Further, cell-specific knockout mice offer the opportunity to specify the cellular source of EVs. Should this systemic relevance prove to be the case, the development of novel treatment options (e.g., pathoblockers interrupting this molecular mechanism or exosome-targeting could be of high relevance to overcome disease severity and systemic inflammation).

## 4. Materials and Methods

### 4.1. Antibodies, Chemokines and Inhibitors

R&D System (Wiesbaden, Germany): Mouse monoclonal antibody against human ADAM10 ectodomain (clone 163003); mouse monoclonal IgG2B isotype control (Clone 20116); goat polyclonal anti-Mouse IgG (H + L) secondary antibody (Alexa Fluor^®^ 647). Mouse monoclonal antibody against human CD9 (MM2/57); Rabbit polyclonal antibody against human ADAM10 C-terminus was obtained from Invitrogen (Frankfurt, Germany), rabbit polyclonal antibody against human GAPDH from Santa Cruz Biotech (Dallas, TX, USA). Rabbit polyclonal antibody against beta actin from Abcam (Cambridge, UK). Mouse monoclonal antibody against flotillin-1 (Clon 18); mouse monoclonal antibody against human E-cadherin C-terminus from BD Biosciences (Heidelberg, Germany). The small molecule inhibitor for ADAM10 GI254023X was from Merck Millipore (Darmstadt, Germany), and human CCL2 was from Peprotech (Rocky Hill, NJ, USA).

### 4.2. Bacteria Preparation

*P. aeruginosa* (PA103 strain, cytotoxic control strain) were preserved in frozen stock of 20% glycerol in Todd-Hewitt-Bouillon (THB) medium (Roth, Karlsruhe, Germany) at −80 °C. For initial growth, *P. aeruginosa* was streaked out on blood sheep agar plates and incubated overnight (oN) at 37 °C and 0% CO_2_. The next day, a liquid culture in THB medium was started from the fresh plate and incubated overnight at 37 °C at 150 rpm. On day 3, a new culture was inoculated with the oN culture to an optical density at 600 nm (OD_600_) of 0.05 and incubated as outlined above for 3 h until bacteria reached the exponential phase. The *P. aeruginosa* cultures were centrifuged at 6,000 *g* for 5 min, resuspended in PBS, and used for cell stimulation at a in multiplicity of infection (MOI) of 5.

*S. pneumoniae* (R6 strain) also were kept as a frozen stocks of 20% glycerol in THB medium at −80 °C. *S. pneumoniae* were cultured with a starting OD of 0.05 in THB medium at 37 °C and 5 % CO_2_ for 4 h followed by a subsequent centrifugation at 4,000 *g* for 10 min. After centrifugation, *S. pneumoniae* were resuspended in PBS and used for cell stimulation at MOI 5.

For heat in-activation of *P. aeruginosa*, bacteria were prepared as mentioned earlier and incubated in the heatblock for 40 min at 70 °C [30]. Inactivation of bacteria was checked by CFU formation, integrity of bacterial particles was observed by microscopy.

### 4.3. Cell Culture and Cell Stimulation

A549 and THP-1 cells were cultured in DMEM and RPMI1640, respectively, supplemented with 10% (fetal calf serum) FCS as described [57]. Human small airway epithelial cells (HSAEpC) were from PromoCell (Heidelberg, Germany) and were cultured in MV2 medium (Promocell, Heidelberg, Germany) as described by the manufacturer. Cells were grown until confluency with a fully supplemented medium for 48 h. For inhibitor pre-incubation, cells were washed with PBS and received serum-free medium with GI254023 (10 μM) for 30 min.

### 4.4. Lentiviral Transduction

ADAM10 short hairpin RNA (shRNA) was loaded into the lentiviral expression vector pLVTHM (Addgene plasmid 12247) targeting ADAM10 mRNA sequences of gacatttcaacctacgaat (ADAM10-KD1) and acagtgcagtccaagtcaa (ADAM10-KD2). In addition, A sequence of ccgtcacatcaattgccgt served as scramble control (scr) [58]. HEK293T cells were used to produce the lentiviral particles by co-transfection with 12.5 µg of the desired pLVTHM-plasmid (additionally coding for GFP), 8.13 µg of psPAX2 (plasmid 12260, Addgene) and 4.37 µg of pMD2.G (plasmid 12259, Addgene) using jetPEI 2 µL/µg of DNA (Polyplus transfection, Illkirch, France) as a transfection reagent. The supernatant was changed after 24 h and the lentiviral particles were resuspended in PBS after being concentrated by centrifugation at 26,000 *g* for 2.5 h after 72 h of the transfection. For transduction, 2 × 10^5^ A549 cells were seeded into six-wells plate, and the concentrated lentivirus was added after 24 h in a polybrene supplemented medium (4 µg/mL) to enhance the transduction efficiency. Transduction efficiency (number of GFP positive cells) was checked by fluorescence microscopy or flow cytometry, knockdown efficiency by Western blot. Cells were expanded and used for experiments within one week after transduction.

### 4.5. Flow Cytometric Analysis

A549 cells and HSAEPC were detached using accutase (Sigma-Aldrich, Munich, Germany) and resuspended in FACS buffer (PBS supplemented with 0.2% BSA). All steps were carried out at 4 °C. The cells were analyzed for the surface expression of ADAM10 by incubation with mouse monoclonal antibodies against human ADAM10 (1 µg/mL) as well as for isotype control (1 µg/mL) in parallel for 1 h followed by incubation with APC-conjugated anti-mouse antibody (5 µg/mL) for 45 min as described [58]. The fluorescence signals were detected by FACS analysis (Sony SH800, Sony, Berlin Germany) and analyzed with FlowJo 10.6.2 software (Tree Star, Inc., Ashland, OR, USA). Dead cells and debris were excluded by forward and side scatter gating. The mean fluorescence values of the same cells number were quantified for each sample, and the signal intensity of the isotype controls were subtracted before comparison.

### 4.6. Western Blot Analysis

Cultured cells were resuspended in 200 µL lysis buffer (20 mM Tris·HCl, 150 mM NaCl, 1% Triton X-100, 1 mM EDTA, 1 mM Na3VO4, 1 mM PMSF, 10 mM 1,10- phenanthroline monohydrate) supplemented with 1× Complete Inhibitor (Roche Diagnostics Deutschland GmbH, Mannheim, Germany) and incubated at 4 °C for 10 min. Cell lysates were centrifuged at 16,000 *g* for 15 min at 4 °C and the supernatant were assigned for protein quantification using a commercial Bicinchoninic acid assay (BCA) kit (Thermo Fisher, Karlsruhe, Germany) following the manufacturer’s protocol. 60 µg of proteins for each sample were heated in SDS buffer (250 mM Tris HCl (pH 6.8), 50% glycerol, 10% SDS, 0.1% bromophenol blue, and 5% *β*-mercaptoethanol) under reducing condition at 60 °C for 30 min and separated by SDS-PAGE using 10% Tris-glycine gels. Proteins were then transferred onto nitrocellulose membrane (Amersham Protran Premium 0.45 NC, GE Healthcare Life Sciences, Freiburg, Germany) and blocked with 5% non-fatty milk in Tris-buffered saline with 0.05% Tween for 1 h followed by an overnight incubation with the desired primary antibody at 4 °C. Consequently, the membrane incubated with the secondary antibody for 1 h, followed by addition of chemiluminescence substrate (PerkinElmer, Waltham, MA, USA) to detect the chemiluminescence signals using luminescent image analyzer LAS3000 (Fujifilm, Tokyo, Japan). Densitometric quantification was performed by quantifying each band of interest and subtracting the background using AIDA Image Analysis software 4.27.039 (Elysia-raytest, Straubenhardt, Germany). Constant square sizes were used to ensure equality between the samples.

### 4.7. Alkaline Phosphatase Assay

A549 cells were transfected with plasmid encoding N-terminally alkaline phosphatase (AP)-tagged betacellulin using Lipofectamine™ 3000 (Thermo Fisher, Karlsruhe, Germany) as a transfection reagent as described earlier in details and seeded in 12-well plates at a density of 3 × 10^5^ [25]. After stimulation (for treatment see figure legends), 100 µL of cell culture supernatant or cell lysates (1% Triton-X100 in TBS supplemented with 1x Complete Inhibitor (Roche Diagnostics Deutschland GmbH, Mannheim, Germany) were transferred to 96-well plates and supplemented with 100 µL of a 2 mg/mL solution of the alkaline phosphatase substrate 4-nitrophenyl phosphate (40 mM Tris-HCl, 40 mM NaCl, 10 mM MgCl2, PH: 9.5). Subsequently, AP activity was determined in the cell lysate and the supernatant by measuring the absorption at 405 nm every 1.5 min for 2.5 h at 37 °C using Genios fluorescence reader (Tecan, Grödig, Austria) as described [25]. The final activity was determined by calculating the ratio of the activity in the supernatant to the total activity (lysate and supernatant).

### 4.8. Protein Permeability Assay

A549 cells were seeded on collagen G-coated polycarbonate trans-well filters with 5 µm pores (Corning, Amsterdam, Netherlands) and grown to confluence (checked by parallel seeding of the same cell number in a 96-well). In contrast to polarized primary cells, A549 cells do not form a barrier for pharmaceutical substances/small molecules (excluding transepithelial electrical resistance (TEER) measurements) but are suitable to study changes in paracellular and transcellular protein as well as permeability for cells (e.g., monocytes). TRITC-dextran was used to monitor paracellular flow indicating loss of barrier integrity, whereas FITC-albumin was used to monitor the sum of paracellular and transcellular permeability changes (referred to total permeability).

After stimulation (for treatment see figure legends), the cell culture medium in the upper chamber was replaced by 70 kDa TRITC-dextran and FITC-albumin suspension (Sigma-Aldrich Chemie GmbH, Taufkirchen, Germany, 1 mg/mL and 0.25 mg/mL, respectively in PBS with 0.2% BSA). After 90 min incubation at 37 °C and 5% CO2, permeability was analyzed by measurement of the fluorescence intensity in the lower chamber at 535-nm excitation and 595-nm emission wavelength for TRITC-dextran and 485-nm excitation and 535-nm emission wavelength for FITC Albumin using the Genios fluorescence reader (Tecan, Grödig, Austria). The results were calculated as the percentage of diffused dextran and albumin in the lower wells relative to a background insert without any cells.

### 4.9. Transepithelial Migration

A549 cells were seeded on 5 µm transwell inserts and grown to confluence. After stimulation (for treatment see figure legends), the lower chamber was filled with 600 µL RPMI/0.2% BSA with or without human CCL-2 (3 nM) followed by an addition of 2 × 10^5^ THP-1 cells to the upper chamber for 2 h. The transmigrated THP-1 cells in the lower chamber were quantified by lysing the cells by addition of 0.1% Triton-X100 (final concentration, with flushing of the membrane’s lower side) and measurement of endogenous β-glucuronidase activity [59]. This method was used instead of cell counting to avoid falsification by cell adhesion in the lower well. The number of transmigrated cells was determined using a serially diluted standard of a defined cell number in parallel. The absorption was measured at 405 nm using Genios fluorescence reader (Tecan, Grödig, Austria). Data were calculated as percentage of transmigrated cells relative to the migrated cells in an empty transwell (no cells).

### 4.10. Wound Closure Assay (Scratch Assay)

For live-cell analysis of scratch-induced wound closure, 2 × 10^4^ cells per well were seeded on collagen G (40 µg/mL) (Biochrom GmbH, Berlin, Germany) coated 96-well plates near confluence and allowed to grow overnight in standard medium. At confluence, cells were pre-treated for 2 h with mitomycin (5 µg/mL) to inhibit cell proliferation followed by 3 times washing with PBS. Subsequently, a defined scratch was performed in each well using the certified BioTec autoscratch (BioTek, Highland Park, Winooski, VT, USA) for 96-well plates ensuring an equal scratch of around 1.2 cm^2^ in each well. The medium was removed and 100 µL standard medium were added to the wells. The closure of the wounded area was monitored using the Lionheart (FX) Automated Microscope system by taking images of each well every 2 h over a period of 24 h. The reduction of wound width was determined over time using the Gen5 software version 3.05.11. For accurate measurement of control cells, wound closure was determined after 14 h. Therefore, an automated primary mask that quantifies the area in the image containing cells was used to quantify the progression of cell migration. Data were expressed as percentage of scratch closure.

### 4.11. Exosome Preparation

To prepare exosomes from A549 cells, 2 × 10^7^ cells were cultured in serum free medium for 2 h followed by the indicated treatments. Subsequently, the medium was collected and centrifuged for 10 min at 300 *g*, followed by 20 min at 1000 *g* and 30 min at 10,000 *g*. After each of the three centrifugations, the pellets were lysed in SDS buffer and the supernatant was subjected to the next centrifugation step. The resulting supernatant was filtrated through a 0.22 μm membrane filter and extracellular vesicles were collected by centrifugation at 100,000 *g* for 1 h at 4 °C using a Beckman rotor Type Ti50.2 (Beckman Coulter GmbH, Krefeld, Germany). The pellet was washed with a high volume of ice-cold PBS (10 mL) and sedimented again at 100,000 g for 1 h. Vesicles were directly dissolved in SDS-sample buffer for Western blot analysis, used for cell stimulation (for AP-Assay) or further fractionated by gradient centrifugation for pure exosome preparation. In the latter case, vesicles were resuspended in PBS and loaded on a continuous gradient comprising layers of 2, 1.3, 1.16, 0.8, 0.5 and 0.25 M sucrose. After centrifugation at 100,000 *g* for 16 h at 4 °C (Beckman Ti50.2 rotor), six 1 mL fractions were collected from the top of the gradient. For each fraction, the density was measured and 1 mL of each fraction was centrifuged at 150,000 *g* for 4 h at 4 °C using a Beckman TLA-55 rotor. The sediment was analyzed by Western blotting.

### 4.12. Measurement of Cytoplasmic Ca^2+^ by Fura-2

A549 cells were seeded at a density of 2 × 10^5^ cells on poly-L-lysine (Sigma, Steinheim, Germany) coated glass coverslips and cultured overnight reaching 70 % confluence. The cells were stimulated for 4 h with PBS or ExoA (100 ng/mL). After 4 h, the cells were loaded with 5 μM Fura-2 AM (Invitrogen by ThermoFischer Scientific, Eugene, OR, USA) for 45 min at 37 °C. After loading, cells were washed with Tyrode’s solution (140 mM NaCl, 4 mM KCl, 1 mM MgCl_2_, 10 mM HEPES and 10 mM D-Glucose, +/− 2 mM CaCl_2_ as indicated in each section, pH 7.4 adjusted with NaOH). Consequently, coverslips were then placed into a circular open-bottom chamber supplemented with 300 μL Tyrode’s solution and mounted onto the stage of the monochromator equipped (Polychrome V, TILL Photonics, Germany) inverted microscope (Axiovert S100, Carl Zeiss, Jena, Germany) at 21 °C. Every 2 s, Fura-2 was alternately excited (0.5 Hz) at 340 and 380 nm for 20 ms and the emitted fluorescence was recorded with a cooled charge-coupled device (CCD) camera (Imago, TILL Photonics). After background correction, ratio images were calculated from 340 and 380 nm pictures. The same amount of cells per sample were marked as regions of interest (ROI) and ratio changes of single ROIs were automatically plotted versus time. Monochromator, camera acquisition, and analysis were controlled by TillvisION software 2.7.0.16 (TILL Photonics, Kaufbeuren, Germany) and results are shown as ratio F340/F380.

### 4.13. Statistical Analysis

Quantitative data are shown as mean +/− SD from three independent experiments/cell unless indicated otherwise. Statistical analysis was performed using GraphPad PRISM 9.0 (GraphPad Software, La Jollla, CA, USA). For details see figure legends.

## Data Availability

The data presented in this study are available on request from the corresponding author.

## Supplementary Materials

The following supporting information can be downloaded at: https://www.mdpi.com/article/10.3390/ijms23031259/s1.
