# Supplementary material for: Pseudomonas aeruginosa Triggered Exosomal Release of ADAM10 Mediates Proteolytic Cleavage in Trans
Source: Int J Mol Sci. 2022 Jan 23;23(3):1259. doi: 10.3390/ijms23031259 (PMC8835980; doi:10.3390/ijms23031259)
Supplement: Supplementary file 1 [file ijms-23-01259-s001.zip › ijms-1558316-supplementary.pdf]

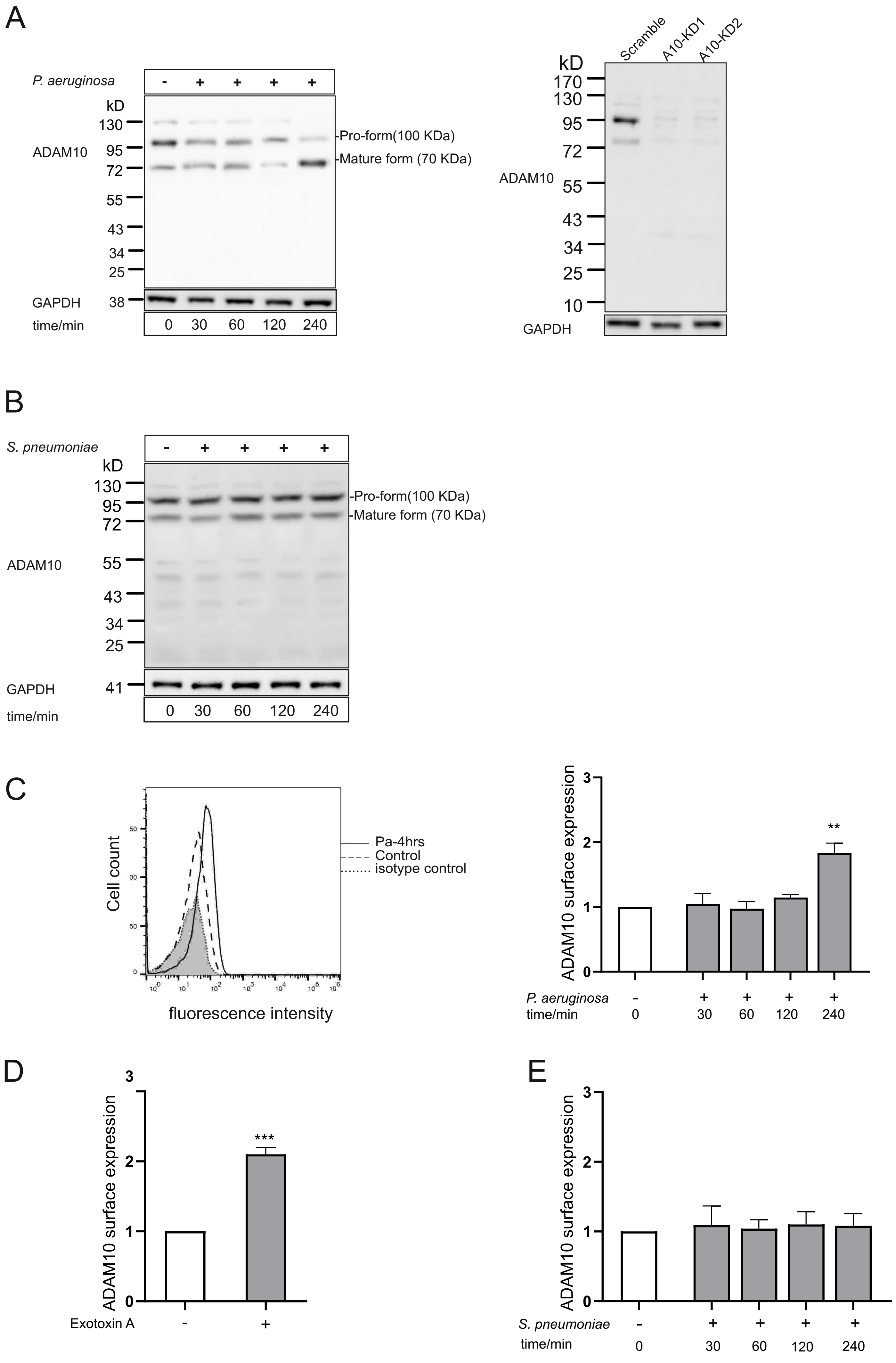

A
P. aeruginosa
- + + + +
kD
130
95
72
55
43
34
25
ADAM10
-Pro-form(100 KDa)
-Mature form (70 KDa)
GAPDH 38
time/min 0 30 60 120 240
Scramble
A10-KD1
A10-KD2
kD
170
130
95
72
55
43
34
25
10
ADAM10
GAPDH
B
S. pneumoniae
- + + + +
kD
130
95
72
55
43
34
25
ADAM10
-Pro-form(100 KDa)
-Mature form (70 KDa)
GAPDH 41
time/min 0 30 60 120 240
C
Cell count
fluorescence intensity
Pa-4hrs
Control
isotype control
ADAM10 surface expression
3
2
1
0
**
P. aeruginosa - + + + +
time/min 0 30 60 120 240
D
ADAM10 surface expression
3
2
1
0
***
Exotoxin A - +
E
ADAM10 surface expression
3
2
1
0
S. pneumoniae - + + + +
time/min 0 30 60 120 240

Figure S1 Control experiments and regulation of ADAM10 in HSAEpC.

In A, the uncropped version of the Western blot shown in Figure 1A (left) is shown in parallel to a Western blot for knockdown control (right). A549 were transduced with lentivirus encoding shRNA against ADAM10 for knockdown (KD) (A10-KD1 or 10-KD2) or an unspecific shRNA (scramble, scr). Notably, the band of ADAM10 pro-from (100 kDa) and mature form (70 kDa) are absent after knockdown serving additionally as proof-of-concept for the bands selected for intensity quantification. (B) Uncropped version of the Western blot shown in Figure 1C. (C-E): HSAEpC were grown to confluence and either left unstimulated or infected with *P. aeruginosa* in C (MOI 5), stimulated with ExoA (100 ng/ml) in D, or infected with *S. pneumoniae* (MOI 5) in E. In C and E, samples were taken after an incubation time of 30, 60, 120 or 240 min. In D, samples were probed after 4 hours. ADAM10 surface expression was investigated by surface staining with an N-terminal antibody against ADAM10 (1 µg/ml) and an APC-coupled secondary antibody (5 µg/ml) and subsequent flow cytometric analysis (quantification as mean fluorescent intensity). The values of the adequate isotype control were subtracted followed by normalization to the unstimulated cells. A representative histogram is shown in C. Quantitative data are shown as means + SD of three independent experiments. Asterisks indicate significance difference to the control calculated using two tailed two samples t-test (**p < 0.01, ***p < 0.001).

A

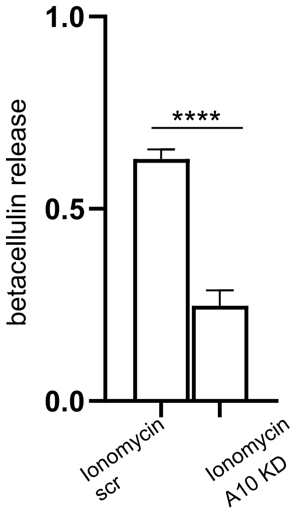
1.0
0.5
0.0
betacellulin release
****
Ionomycin scr
Ionomycin A10 KD

Figure S2 Control experiment activity measurement. (A)

A549 were transduced with lentivirus encoding shRNA against ADAM10 for knockdown (KD) (A10-KD1 or 10-KD2) or an unspecific shRNA (scramble, scr). These cells were transfected with a plasmid encoding for alkaline phosphatase (AP)-coupled betacellulin (AP-BTC) and seeded at equal density. Cells were stimulated with 1 µM ionomycin for 1 hour. Finally, AP activity was determined in the cell lysate and supernatant to quantify the relative betacellulin cleavage and release. Data are shown as means + SD of three independent experiments. Asterisks indicate significance among treated cells calculated using 1-way ANOVA and Tukey post-test (****$p < 0.0001$).

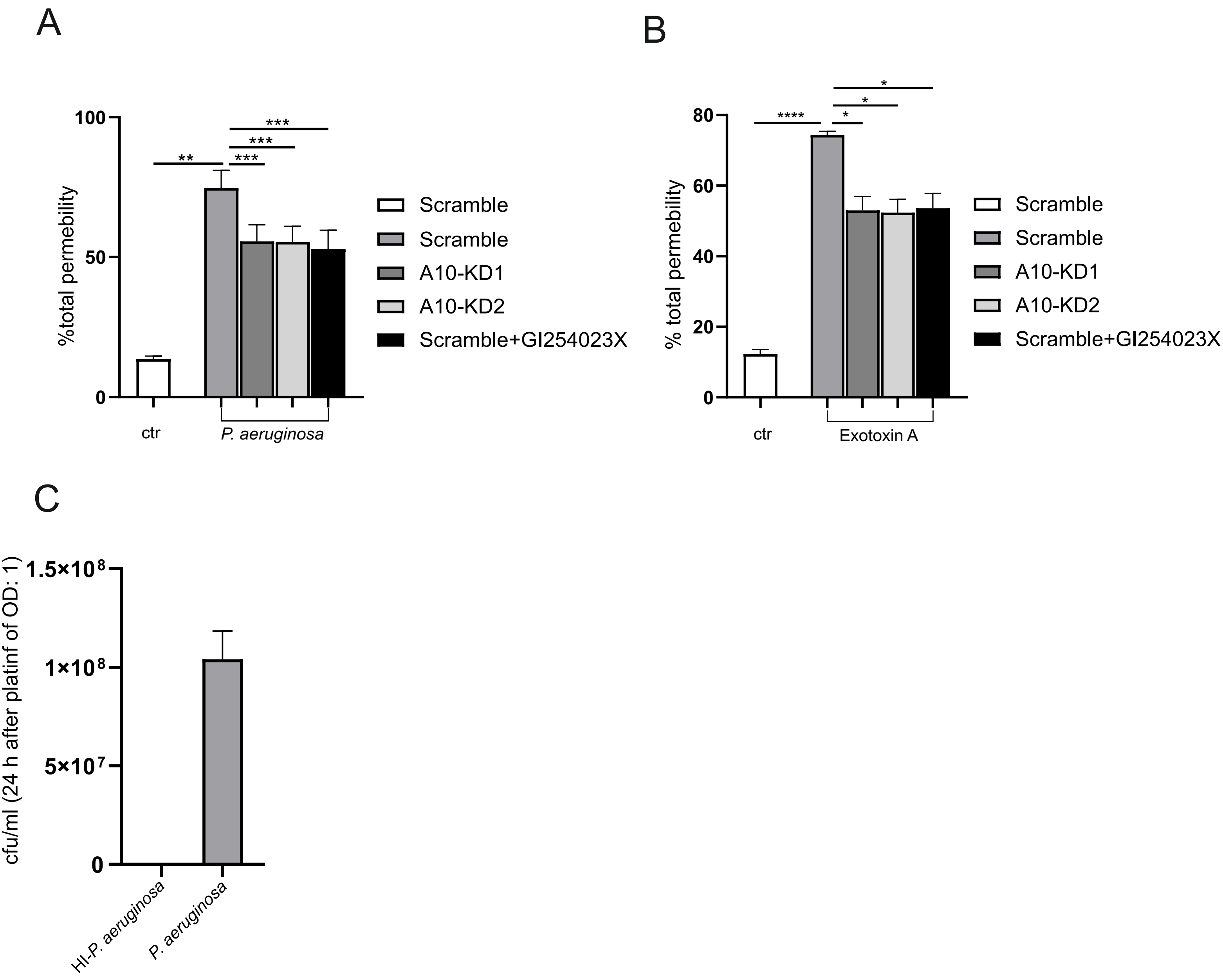
A
%total permebility
100
50
0
**
***
***
***
ctr
P. aeruginosa
Scramble
Scramble
A10-KD1
A10-KD2
Scramble+GI254023X
B
% total permebility
80
60
40
20
0
****
*
*
*
ctr
Exotoxin A
Scramble
Scramble
A10-KD1
A10-KD2
Scramble+GI254023X
C
cfu/ml (24 h after platinf of OD: 1)
1.5×10^8
1×10^8
5×10^7
0
HI-P. aeruginosa
P. aeruginosa

Figure S3 Measurement of total permeability and control of heat-inactivation. (A/B): A549 were transduced with lentivirus encoding shRNA against ADAM10 for knockdown (KD) (A10-KD1 or 10-KD2) or an unspecific shRNA (scramble, scr) as indicated in the graph legend. Cells were grown in transwells until confluence. Cells were pre-incubated with 0.1 % DMSO (vehicle control) or with ADAM10 inhibitor GI254023X (10 µM, black bars). Cells were either left unstimulated or infected with *P. aeruginosa* in A (MOI 5 for 4 hours) or stimulated with ExoA in B (100 ng/ml for 4 hours). Subsequently, the cell culture medium in the upper chamber was replaced by 70-kDa TRITC-dextran and FITC-albumin suspension in PBS supplemented with 0.2 % BSA, and the permeability was measured by TRITC dextran and FITC albumin diffusion into the lower wells. Total permeability (paracellular and transcellular) is shown as percentage, calculated in relation to the background empty transwell (100%). (C) *P. aeruginosa* cultures were incubated in the heatblock for 40 min at 70 °C and compared to non-treated cultures. Cultures were adjusted to OD 1, plated on agar plates, grown for 24 hours and used for determination of the original colony forming units (CFU). Data are shown as mean + SD of three independent experiments. Asterisks indicate significance among treated cells calculated using 1-way ANOVA and Tukey post-test in A and B and two tailed two samples t-test for C (*p < 0.05, **p < 0.001, ***p < 0.001, ****p < 0.0001).
